# Inhibition Mechanism of Water-Soluble Chitosan–Curdlan Composite Coating on the Postharvest Pathogens of *Serratia marcescens* and *Pseudomonas syringae* in Cherry Tomatoes

**DOI:** 10.3390/microorganisms12061149

**Published:** 2024-06-05

**Authors:** Kejing Yan, Kunyu Liu, Jiaqi Chang, Ziyu Jing, Jiasi Li, Youwei Yu, Shaoying Zhang

**Affiliations:** College of Food Science, Shanxi Normal University, Taiyuan 030031, China; 18835735152@163.com (K.Y.); liukunyu0622@163.com (K.L.); jingziyu010802@163.com (Z.J.); lijiasi2002@163.com (J.L.)

**Keywords:** *Serratia marcescens*, *Pseudomonas syringae*, chitosan, composite coating, inhibition mechanism

## Abstract

Cherry tomatoes, a very popular fruit, are highly susceptible to microbial infestation, which cause significant economic losses. In order to preserve cherry tomatoes better, we treat them with a Chitosan (CTS) and Curdlan (CUR) composite coating. The lowest inhibitory concentration of CTS/CUR composite coating on *Serratia marcescens* and *Pseudomonas syringae*, the growth curves, and the changes of the cell lysis rate were determined to explore the inhibitory mechanism of CTS/CUR composite coating on *Serratia marcescens* and *Pseudomonas syringae* and the microscopic morphology of *Serratia marcescens* and *Pseudomonas syringae* was observed using scanning electron microscopy at the same time. The results showed that the CTS/CUR composite coating could effectively inhibit the growth of *Serratia marcescens* and *Pseudomonas*, and the inhibitory effect reflected the concentration-dependent characteristics. The electron microscopy results indicated that the inhibition of *Serratia marcescens* and *Pseudomonas syringae* by the CTS/CUR composite coating might originate from its disruptive effect on the cell wall and cell membrane of the bacterium.

## 1. Introduction

In recent years, food safety problems caused by microbial contamination have been reported at home and abroad. Microorganisms are diverse and multiply rapidly, so food spoilage caused by microorganisms is difficult to control [1]. In fruits and vegetables, microbial problems are even more prevalent and could easily cause economic losses to fruit and vegetable farmers. Cherry tomatoes, as an extremely popular fruit worldwide, have attracted a lot of attention in terms of storage and preservation. Cherry tomatoes are susceptible to many plant pathogenic diseases, and the growth of microorganisms is often inhibited by adding preservatives in the food industry [2]. Chemical synthetic preservatives are now more widely used, but people are beginning to worry about their potential hazards. Tomato bacterial spot disease was also known as tomato bacterial leaf spot or blotch disease, and its pathogen is *Pseudomonas syringae* [3]. These bacteria damage the fruit, leaves, stems, flowers, and petioles of tomato. Once a tomato fruit is infected, the flesh near the spot is slightly sunken. The spot is surrounded by a black color, and the middle is light in color and slightly concave [4]. The soft rot disease of fruit and vegetable usually caused by bacterial infection [5]. *Serratia marcescen* is one of pathogens that cause soft rot disease [6]. As for tomato, its soft rot disease starts from the location of the wound on the whole branch, then extends inwards, and finally rots the pulp. After the pathogen invades, it secretes pectinase to dissolve the intermediate layer between the host cells, separate the cells, and cause soft rot [7].

Edible coating preservation technology can effectively inhibit the invasion of postharvest pathogenic microorganisms and reduce mechanical damage to the surface of fruits and vegetables. Coating preservation technology has been applied in many postharvest fruits, such as apple, grape, citrus, etc., [8,9]. Chitosan is a widely used coating material for fruits and vegetables. It has excellent antibacterial or antifungal effects, inhibiting postharvest pathogenic microorganisms to some extent [10]. Curdlan, a linear structure composed of D-glucose via β-1, 3-glucoside bond, is a novel extracellular polysaccharide [11]. However, as a natural composite biological preservative, chitosan and curdlan gum are rarely reported as being used to prepare bacteriostatic coating [12]. The development of this new composite coating could be achieved through sustainable production, making it a potential environmentally friendly material for fruit preservation. In our previous experiment, we prepared and characterized CTS/CUR-composite-coated films with different ratios. The results show that the 1:1 CTS/CUR composite coating demonstrated superior preservative effects and had the potential to significantly extend the shelf life of cherry tomatoes; as such, it could be applied in fruits and vegetables preservation in the future [13].

In this paper, the *Serratia marcescens* and *Pseudomonas syringae* of spoilage bacteria in fruits and vegetables were taken as antibacterial objects. The growth curve, extracellular relative conductivity, DNA, and protein leakage changes before and after the interaction of the composite coating with pathogenic bacteria were measured; the effects of composite coating on the cell membrane and intracellular molecules of pathogenic bacteria were explored as well. Our research explored the antibacterial mechanism and mode of chitosan and curdlan gum composite coating, providing certain reference for the application of prepared composite coating to preserve fruits and vegetables.

## 2. Materials and Methods

### 2.1. Materials

Water-soluble chitosan (chitosan amino salt-forming type, generally lactate; molecular formula: (C_9_H_17_NO_7_)_n_) was obtained from Ao Kang Biotechnology Co., Ltd., Jinan, China). Curdlan was obtained from Green City Biology Co., Ltd. (Guangzhou, China). Glycerol was purchased from Fengchuan Chemical Reagent Technology Co., Ltd. (Tianjin, China). *Serratia marcescens* and *Pseudomonas syringae* were used as test strains. *Serratia marcescens* was isolated and *Pseudomonas syringae* (CGMCC1.3156) was purchased from the Chinese Strain Preservation Center. Cherry tomatoes were obtained from local markets (Taiyuan, China), and the fruits were bright in color and not mechanically damaged.

### 2.2. Preparation of Coatings

The coatings were prepared according to Yu Y [13]. At 60 °C, the solid powders of CTS and CUR solid were mixed using deionized water in 1:1 (*w*/*v*). Then, 0.1 mL of glycerol was added into 100 mL coating solution as a plasticizer. The mixture was fully stirred for 40 min at 2000 r/min using a magnetic stirring pot (SHJ-6A, Changzhou Jintan Liangyou Instrument Co., Ltd., Changzhou, China) until there were no particles in mixture. Then, the mixture was degassed until there were no bubbles in the solution using a vacuum pump (2XZ, Zhejiang Huangyan Tianlong Vacuum Pump Co., Ltd., Taizhou, China). Afterwards, the solution was left to stand for 8 h. Then, 35 mL of solution was cast on a 10 cm × 10 cm acrylic film and dried to form a coating at 30 °C. The coating was uncovered and stored in a dryer for related properties determination.

### 2.3. Isolation of Serratia marcescens

Three tomatoes with rot symptoms which matched *Serratia marcescens* were randomly selected, and samples were randomly taken using a sterile perforator. A total 3 g of flesh tissue samples from each variety were placed into the sterile mortar, and 0.85% NaCl solution was added. The mixtures were ground thoroughly to make the samples homogeneous. Then, 1 mL of homogenate was gradient diluted to concentrations ranging from 10^−1^ to 10^−7^ cfu/mL according to the 10 fold dilution method, and then the diluted solution was coated in LB medium and inverted at 37 °C for 24 h to observe the growth of the plate. A certain number of single colonies were selected for cultivation based on the colony morphology, and the pure culture and sterile glycerol mixture was stored in an ultra-low temperature refrigerator at −80 °C for preservation and retention. The remaining bacterial solution was subjected to 16S rDNA sequence amplification, referring to the method of Liu J [14]. The qualified PCR amplification products were sent to the company for sequencing after electrophoresis detection, and the positive sequence obtained from sequencing was compared and screened to identify *Serratia marcescens*.

### 2.4. Cultivation Methods of Serratia marcescens and Pseudomonas syringae

In all experiments, the inoculum used in the measurement was obtained from overnight culture grown on the NA slope at 37 °C. We diluted the culture in an 85% sterile saline solution to a final concentration of approximately 10^8^ CFU/mL and adjusted the turbidity according to the McFarlane standard tube. The final concentration of the inoculum in the medium used for the antimicrobial assay was approximately 10^7^ CFU/mL.

### 2.5. Inhibition Effect of Composite Coating on Bacteria

The antibacterial zone was determined using the filter paper method, with slight modifications to the Oxford Cup method described in reference [15]. The bacterial strains were activated on sterile NB agar plates to prepare bacterial solution, then diluted it to 1 × 10^6^–10^7^ CFU/mL. Then, 100 μL of bacterial solution was added to each plate and applied evenly with an applicator. The sterilized filter paper was gently placed in the center of each plate, and then, at a concentration of 1 mg/mL, 100 μL of the CTS, CUR, and CTS/CUR composite coating solutions and 100 μL of 75% ethanol were added into each plate. Plates were incubated at 37 °C for 24 h. Each group was repeated three times and observed their antibacterial activity.

### 2.6. Determination of MIC and MBC of Bacteria Treated with Composite Coating

The determination of minimum inhibitory concentration (MIC) and minimum bactericidal concentration (MBC) were carried out according to the reference multiplicative dilution method with slight modification [16]. CTS, CUR, and composite coating solutions were used as experimental groups, and the three drugs were doubly diluted into a series of concentration gradients, which were added to sterilized test tubes containing 5 mL of culture medium without the drugs as a negative control. Finally, 50 μL of diluted bacterial liquid was added at a concentration of 1 × 10^6^–10^7^ CFU/mL. Then, the test tubes were placed in 37 °C shaking bed culture for 24 h and observed. The liquid in the test tube was clarified, and the concentration of the drug corresponding to the sterile growth was taken as the minimum inhibitory concentration (MIC) of the bacteria. The incubation was continued for 24 h, the liquid in the test tube was clarified, and the concentration of the drug corresponding to the sterile growth was taken as the minimum bactericidal concentration (MBC) of the bacteria.

### 2.7. Effect of Composite Coating on Bacterial Growth Curve

Tests were carried out by the method described in reference [17]. The test was carried out based on the diameter of the antibacterial zone and the results of MIC and MBC. Several conical flasks containing 50 mL of sterile medium were taken, to which 1 mL of diluted water-soluble chitosan, curdlan, and composite coating solution with concentrations of 1/4 × MIC, 1/2 × MIC, MIC, and 2 × MIC were added, while one conical flask was used as a blank control, and finally the diluted bacterial solution with concentrations of 1 × 10^6^–10^7^ CFU/mL was added to each flask, respectively. Finally, 0.5 mL of diluted bacterial solution with a concentration of 1 × 10^6^–10^7^ CFU/mL was added to each culture bottle, incubated at 37 °C on a shaking table, and the samples were taken at the time points of 0, 1, 2, 4, 6, 8, 10, 12, 14, 16, 18, 20, 22, and 24 h, and the absorbance values were measured at 600 nm. The time-OD curves were plotted.

### 2.8. Cell Morphology by Scanning Electronic Microscopy

The experiment was conducted using the method described in reference [18]. Firstly, *Serratia marcescens* and *Pseudomonas syringae* were cultured in a fresh liquid medium until the logarithmic phase. The cultured liquid was centrifuged at 5000× *g* for 5 min. The organisms were collected and resuspended in a fresh medium. The bacterial concentration was adjusted to 1 × 10^9^ CFU/mL, then the bacterial solution was divided into 100 mL conical flasks, each bottle containing 50 mL of bacterial solution. Then, 1 mL of the drugs with a concentration of 1 × MIC was added, without any substances, as a blank tube, and the conical flasks were incubated on a shaking table at 37 °C. After 8 h of cultivation, a sample of 10 mL was taken and centrifuged at 4 °C for 5 min at 5000× *g*, then washed three times with 0.1 M PBS (PH 7.0), and the washed sample was centrifuged to obtain the bacterial body. Subsequently, 2.5% glutaraldehyde was added so that the bacteria were resuspended in glutaraldehyde and fixed overnight; after fixation, the sample solution was centrifuged at 5000× *g* for 5 min and then washed three times with 0.1 M PBS (PH 7.0) to obtain the bacterial body. Finally, the bacterial body was dehydrated with 30%, 50%, 70%, 90% and 100% ethanol gradient, the supernatant was discarded, and 100 μL of isoamyl acetate was added and mixed well. A drop of isoamyl acetate bacterial solution was dipped into the gun tip and added to the cover glass on the conductive tape. After the isoamyl acetate evaporated and dried, it was sprayed with gold and observed under a scanning electron microscope.

### 2.9. Effect of Composite Coating on Bacterial Cell Membrane Permeability

Tests were conducted using the method described in the reference [19]. Firstly, 5% glucose solution was prepared with fresh double distilled water, and its conductivity was measured, denoted as L0′. Next, 5000× *g* was centrifuged for 5 min to collect the bacterial cells cultured at 37 °C until the logarithmic phase. Then, they were washed three times with 5% glucose and resuspended to make the conductivity of the bacterial solution close to that of 5% glucose. Subsequently, 50 mL of bacterial solution (1 × 10^9^ CFU/mL) and 1 mL of diluted drug were taken to mix, with a concentration set to 0.25 × MIC, 0.5 × MIC, 1 × MIC, 2 × MIC, as a blank control without medication. The conductivity value was measured immediately after mixing and recorded as L1; then, it was placed at 4 °C and incubated on a shaking bed at 120 r/min, and the conductivity values were measured at 0.25, 0.5, 0.75, 1, 2, 4, and 6 h, denoted as L2. Finally, the conductivity of the blank bacterial solution after boiling was measured as L0. The relative conductivity was calculated based on the following formula. The curve of relative conductivity over time was drawn based on the results.

Relative conductivity (%) = (L2 − L1)/(L0 − L0′) × 100%.

### 2.10. Detection of Bacterial Cell Membrane Permeability

The experiment was determined according to the method described in the reference with slight modification [20]: the bacterial fluid that was just cultured to logarithmic stage at 37 °C was removed and transferred to a 50 mL centrifuge tube where it was centrifuged at 5000× *g* for 10 min, then the supernatant was discarded and the precipitated bacterial body was left for spare. It was washed three times with filtered PBS and resuspended, the concentration, of the bacterial body was adjusted to 1 × 10^9^ CFU/mL, 5 centrifugal tubes were taken and the drugs were added to create concentrations of 0, 0.25 × MIC, 0.5 × MIC, 1 × MIC, and 2 × MIC, respectively. In this case, the blank control group (CK) is the group to which the concentration of drug added is 0. The drugs were mixed well and then placed in a shaker incubator for 1 and 2 h. A total of 150 μL of bacterial fluid was taken out at each time point, centrifuged at 5000× *g*, mixed with PBS (PH 7.0), and washed twice, and then 100 μL of PI fluorescence staining solution was added, quickly mixed, and protected from light for 15 min to stain. The bacterial body was obtained by centrifuge, resuspended in microfilm-filtered PBS (PH 7.0) to 100 μL, and mixed thoroughly. Then, 3 μL of the bacterial body was taken and put on a clean slide with a coverslip and pressed gently. The bacterial body was observed under a fluorescence microscope with a fluorescence filter block and pictures were taken to record.

### 2.11. Effect of Composite Coating Solution on Bacterial Cell Leakage

The bacterial suspension, after secondary activation, was collected by centrifugation (8000× *g*, 5 min), washed three times with PBS (PH 7.0), diluted, and treated with three pharmaceuticals at concentrations of 0, 0.25 × MIC, 0.5 × MIC, 1 × MIC, and 2 × MIC, respectively. The treatment without drugs was used as a blank control and placed in a constant temperature incubator at 37 °C for 4 h. The treated bacterial suspension was centrifuged (8000× *g*, 4 min), and its supernatant was taken to determine its absorbance values at 260 nm and 280 nm, respectively; the measured absorbance values represented the content of nucleic acids and proteins in the bacterial suspension [21].

### 2.12. Bacteriostasis Experiment on Cherry Tomatoes

Cherry tomatoes were washed by distilled water and allowed to naturally air-dry. Each group of 50 fruits was treated with CTS, CUR, and composite coatings, respectively, while the control group was treated with sterile water. Each group was impregnated for 30 min and allowed to naturally air-dry. We punched a hole with a width of 2 mm and a depth of 3 mm in the equatorial part, and 10 μL of *Pseudomonas syringae* and *Serrania marcescens* suspensions were inoculated individually. The tomatoes were sealed with a plastic fresh-keeping box and stored at RH 95~98%, 25 ± 1 °C.

### 2.13. Statistical Analysis

Data are presented as mean ± standard deviation. One-way ANOVA and Duncan’s multiple interval test were used in SPSS 18.0, and *p* ≤ 0.05 was considered to indicate statistical significance. Figures were analyzed using origin 2023.

## 3. Results

### 3.1. Separation Results of Serratia marcescens

**Figure 1:**
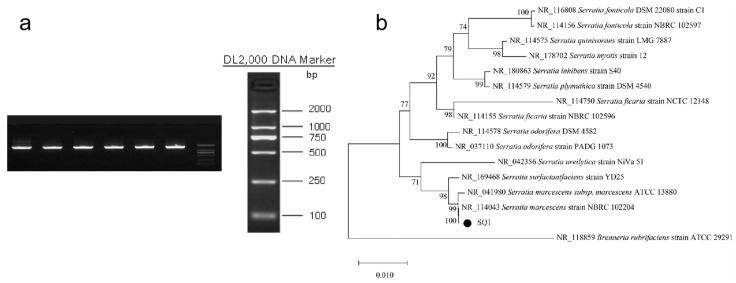
PCR electrophoresis of bacteria (**a**) and phylogenetic tree of 16S rDNA (**b**).

The ribosomal DNA of prokaryotes has three types, 23S, 16S, and 5S, which are directly related to the translation function of proteins [22]. Ribosomes are present in all cellular organisms and have a high degree of sequence conservation, as well as polymorphic regions of high variability within their molecules [23]. Among the three ribosomal molecules, 16S rDNA has been chosen as a yardstick for the evolutionary process of organisms for systematic classification of organisms because it is sufficiently informative and has a moderate sequence size (1.5 K) and is thus the best marker known for phylogenetic studies [24]. Currently, it is generally accepted by bacteriologists that when 16S rDNA sequence homology is higher than 97%, it can be considered homologous within the genus [25]. The extracted strain was amplified by PCR using 16S rDNA primers. As shown in Figure 1, a bright and clear band was detected by electrophoresis, with a molecular size of about 1500 bp. According to the results of strain identification of Shanghai Parsonage Biotechnology Co., Ltd. (Shanghai, China) and BLAST comparison between the sequencing results and the similar sequences on NCBI, it was found that the strain tested is the closest to Serratia marcescens, and the degree of similarity reaches 99.65%.

### 3.2. Inhibition Effect of Composite Coating on Bacteria

**Figure 2:**
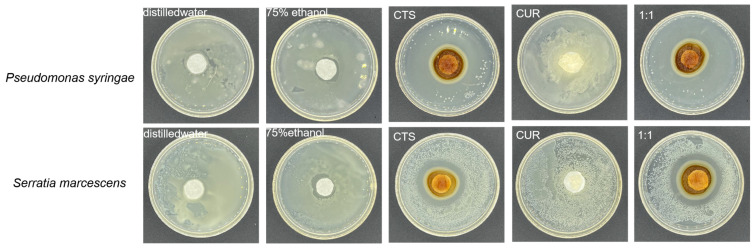
Bacterial inhibition ability of different treatments.

The inhibitory ability of water-soluble chitosan, curdlan, and composite coating solutions on *Pseudomonas syringae* and *Serratia marcescens* were determined using the filter paper method. As shown in Figure 2, the water-soluble chitosan and the composite coating solution showed excellent bacteriostatic effects on *Serratia marcescens* and *Pseudomonas syringae*. However, curdlan showed no inhibitory circle for *Pseudomonas syringae* and *Serratia marcescens*, which indicated that it had no inhibitory effect on them, so curdlan was not determined in the subsequent inhibitory experiments. MIC and MBC results of CTS and the composite coating on *Serratia marcescens* and *Pseudomonas syringae* are shown in Table 1; the MIC of CTS against *Serratia marcescens* was 23.44 μg/mL and the MBC was 46.88 μg/mL, and the MIC against *Pseudomonas syringae* was 18.75 μg/mL and the MBC was 37.50 μg/mL. The MIC of the composite coating solution was 15 μg/mL and the MBC was 30 μg/mL against *Serratia marcescens*, and the MIC was 11.72 μg/mL and the MBC was 23.44 μg/mL against *Pseudomonas syringae.* The composite coating solution had good antibacterial activity against both experimental bacteria, and *Pseudomonas syringae* was more sensitive to it. From the result of MIC and MBC values, we found that the MIC and MBC values of the composite coating solution were lower for the two test organisms, indicating that the inhibitory activity of the composite coating solution was stronger, which was consistent with the results in Figure 2.

### 3.3. Effect of Composite Coating on Bacterial Growth Curve

**Figure 3:**
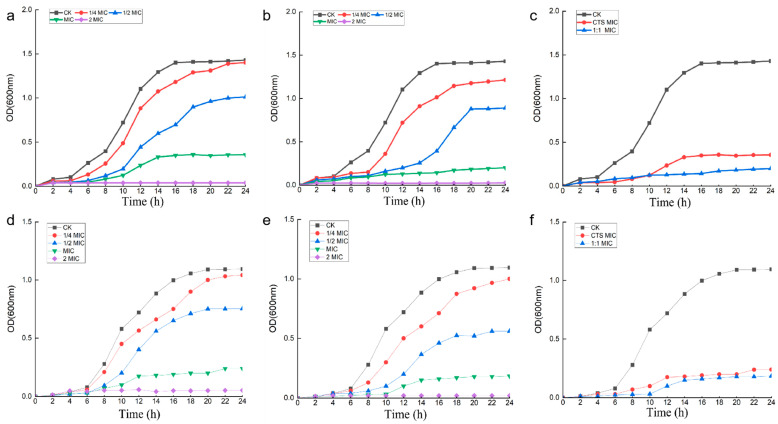
Effect of CTS and composite coating solutions on the growth curves of *Serratia marcescens* and *Pseudomonas syringae*. Among them, (**a**–**c**) is *Pseudomonas syringae*, (**d**–**f**) is *Serratia marcescens*, and from left to right are CTS, composite coating solution (1:1), and MIC, respectively.

Figure 3 showed that the effect of CTS and composite coating solution on the growth curves of *Serratia marcescens* and *Pseudomonas syringae*. Since the concentration of the bacterial suspension is directly proportional to the OD value, the determination of bacterial solution OD value over time can be an indirect reaction to the change rule of the concentration of the bacterial solution over time, and thus we can obtain the growth curve of bacterial cells. As depicted in Figure 3a–c, the OD value began to increase at 6 h, and reached 1.4 from 6 h to 16 h in the blank control group without the addition of drugs; the process of bacterial concentration increased significantly, which is shown as s-type in the graph, and this period is the logarithmic growth period of the bacterial population. In the experimental group with the addition of drugs, in the curve with the addition of drug 1/4 × MIC water-soluble chitosan, it was found that the OD value started to increase gradually from 8 h and reached 1.34 at 22 h. In the curve with the addition of drug 1/4 × MIC composite coating solution, it was found that the OD value started to increase gradually from 8 h and reached 1.21 at 24 h. In the curve of 1/2 × MIC water-soluble chitosan, the OD value increased from 10 h. In the curve of 1/2 × MIC composite coating solution, the OD value was found to increase gradually from 14 h and reached 0.81 at 20 h. In the curve of 1 × MIC water-soluble chitosan, however, the OD value was found to increase gradually from 10 h, and at 14 h, the OD value reached 0.91. In the curve of 1 × MIC composite coating solution, it was found that the OD value increased slowly at 24 h with little magnitude. When both drugs were added at a concentration of 2 × MIC, there was no change in OD values throughout the measurement time of 24 h.

As shown in Figure 3d–f, in the blank control group without the addition of drugs, the OD value began to increase at 6 h, and the OD value reached 1.15 from the beginning of 6 h to 20 h; this process of bacterial concentration increased significantly, which is manifested in the graph as an s-type, and this period is the logarithmic growth period of the bacterial population. In the experimental group with the addition of drugs, it was found that the OD value in the curve with the addition of drug 1/4 × MIC water-soluble chitosan started to increase gradually from 6h and reached 0.93 at 24 h. In the curve with the addition of drug 1/4 × MIC composite coating solution, the OD value started to increase gradually from 8 h and reached 0.86 at 24 h. In the curve of 1/2 × MIC water-soluble chitosan, the OD value started to increase from 8h, and in the curve of 1/2 × MIC composite coating solution, it was found that the OD value started to increase gradually from 10 h, and the OD value reached about 0.5 at 18 h, whereas the OD value increased slowly at 24 h in the curve of 1 × MIC water-soluble chitosan and 1 × MIC composite coating solution with a small magnitude. When the two drugs were added at a concentration of 2 × MIC, the curve of water-soluble chitosan had a slight undulation, while the curve of the composite coated solution did not show any change in the OD values throughout the measurement time of 24 h. As can be seen from Figure 3c,f, the effect of composite coating on the growth curves of both experimental bacteria was greater than that of CTS, and the antibacterial effect of composite coating was better than that of single CTS.

### 3.4. Effect of Composite Coating on the Morphology of Serratia marcescens and Pseudomonas syringae

**Figure 4:**
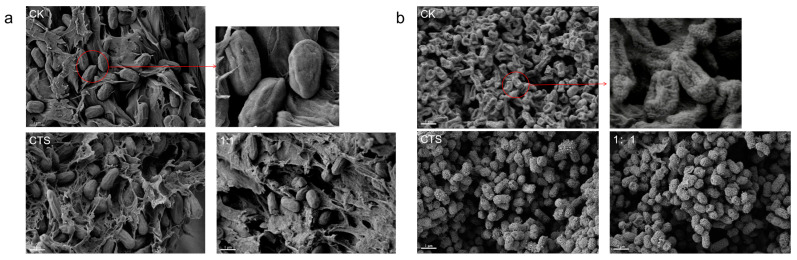
Effect of CTS/CUR composite coating on bacterial morphology of *Serratia marcescens* and *Pseudomonas syringae*. Among them, (**a**) is *Pseudomonas syringae* and (**b**) is *Serratia marcescens*.

The experimental results of *Pseudomonas syringae* and *Serratia marcescens* after composite coating treatment were observed by the electron microscope scanning technique, as shown in Figure 4. Figure 4a clearly shows that the blank control group of *Pseudomonas syringae* was elliptical spherical with wrinkles on the surface; after single CTS and composite coating treatment, the wrinkles became more pronounced. In Figure 4b, it can be seen that the untreated *Serratia mucilaginosa* showed a concave shape with a rough surface and particles, and after single CTS and composite coating treatment, it can obviously be seen that the cells were swollen and the surface particles were distended. In summary, it can be seen that the composite coating treatment did have a direct effect on the cell membrane from the changes in the appearance of the bacterium; obvious changes in its morphology and structure were caused.

### 3.5. Effect of Composite Coating on Cell Integrity of Serratia marcescens and Pseudomonas syringae

**Figure 5:**
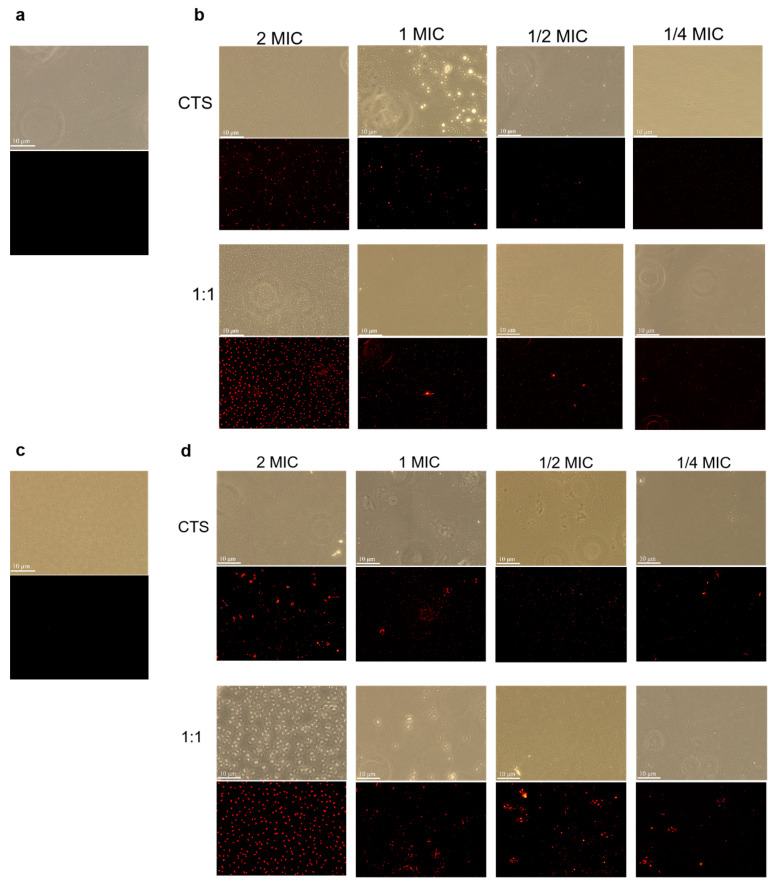
Fluorescence inverted micrographs of *Serratia marcescens* and *Pseudomonas syringae*. Among them, (**a**) *Serratia marcescens* CK; (**c**) *Pseudomonas syringae* CK; (**b**) *Serratia marcescens*; (**d**) *Pseudomonas syringae*.

**Figure 6:**
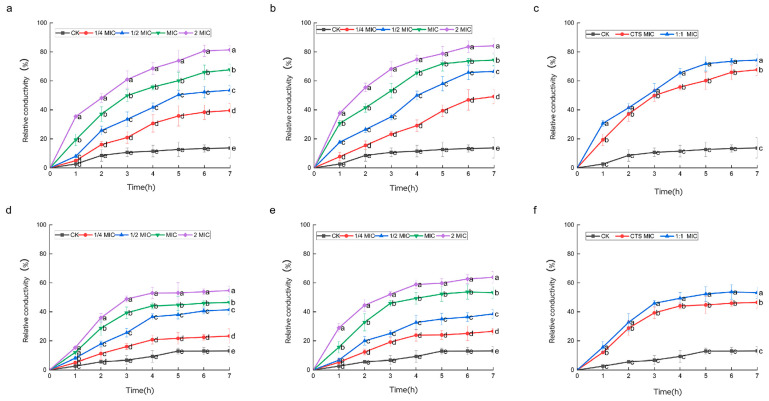
Effect of CTS and composite coating solutions on the relative conductivity of *Serratia marcescens* and *Pseudomonas syringae*. Among them, (**a**–**c**) is *Pseudomonas syringae*, (**d**–**f**) is *Serratia marcescens*, and from left to right are CTS, composite coating solution (1:1), and MIC, respectively. Different lowercase letters indicate remarkable differences between groups at *p* ≤ 0.05.

The PI staining results are shown in Figure 5. If the bacterial cell membrane is intact and PI dye cannot enter the cell under normal conditions, the bacterial nucleus cannot be stained, so fluorescence cannot be observed under a fluorescence microscope. When the cell membrane of the bacterium is damaged, the cell membrane will become loose, and many substances that cannot pass through the bacterium normally can pass through the bacterium freely; at this time, PI also passes very easily through the cell membrane. When PI enters into the cell, it will be combined with the DNA in the cell; at this time, through a certain excitation wavelength, red fluorescence be observed under the fluorescence microscope [26]. The blank group did not display fluorescence (Figure 5a,c), some red fluorescence appeared after 0.5 × MIC treatment (Figure 5b,d), and with the increase in drug concentration, more and more red-fluorescent organisms were stained by PI fluorescence. A large area of fluorescence appeared after 2 × MIC treatment, which indicated that the organisms which had not had the treatment were not stained by PI, while the organisms which were treated by the drug were stained by PI, so that the organisms stained by PI fluorescence were observed under the fluorescence microscope. Under the drug-treated experimental group, there was a positive correlation between the drug concentration and the degree of cell membrane disruption in the two test bacteria. After 2 × MIC treatment (Figure 5b), the composite-coated treatment group appeared to have more red fluorescence than the CTS-treated group, and similar results appear in Figure 6d. Comparing Figure 5b and Figure 6d, *Pseudomonas syringae* appeared to have more red fluorescence than *Serratia marcescens* after the 2 × MIC composite coating treatment. This suggests that CTS and the composite coating solution can act on the cell membranes of *Serratia marcescens* and *Pseudomonas syringae* to increase permeability and that *Pseudomonas syringae* has more severe cell membrane rupture, a result that is also consistent with the relative conductivity results.

### 3.6. Effect of Composite Coating on Cell Membrane Permeability of Serratia marcescens and Pseudomonas syringae

The conductivity method is a simple and effective way to determine whether the bacterial membrane is damaged [27]. When the bacterial membrane is attacked and its selective permeability is altered, the intracellular ions will leak out non-selectively, leading to an increase in extracellular conductivity. The results of relative conductivity changes in CTS and composite coating solution after acting on *Serratia marcescens* and *Pseudomonas syringae* are shown in Figure 6. The relative conductivity of the bacterial suspension in the blank group of the two experimental bacteria showed a small change, and after 1 h, there was a slow increase with a small increase. When the concentration of CTS added is 2 × MIC, the relative conductivity of the bacterial suspension increased by 53.07% and the increase was completed within 3 h. When the action time was prolonged, it was found that the relative conductivity no longer changed significantly. When the concentration of the composite coating solution was added at a concentration of 2 × MIC, the relative conductivity of the bacterial suspension increased by 69.71% and the increase in the relative conductivity kept growing within 7 h. When CTS was added at a concentration of 1 × MIC, the relative conductivity of the bacterial suspension increased by 48.84% at 4 h and no longer changed significantly with time. When the concentration of the composite coating solution added was 1 × MIC, the relative conductivity of the bacterial suspension increased by 57.31%. These results indicate that the addition of CTS and composite coating solution resulted in ionic leakage from the bacteria, which increased the ionic concentration in the culture solution of the bacteria, and that the ionic leakage from the bacteria became more pronounced as the concentration of drug action increased, as evidenced by the higher relative conductivity. When the addition amount reached 2 × MIC, a large portion of the ions in the bacterium had already leaked out, so the relative conductivity was basically unchanged in the subsequent time. Similar to Figure 6d–f, the relative conductivity values of *Serratia marcescens* after the action of different concentrations of CTS and composite coating solutions, shown in Figure 6a–c, were significantly increased; this indicates that CTS and composite coating solutions have the same mechanism of action on *Serratia marcescens*. Among them, the concentration of CTS increased by 62.15% at 1 × MIC and the concentration of the composite coating solution increased by 73.86% at 1 × MIC. However, within the action time, the relative conductivity of the *Pseudomonas syringae* experimental group did not reach a stable value, so that the ion leakage in *Pseudomonas syringae* was not yet finished, probably because *Pseudomonas syringae* is more sensitive, which is in line with the results of the MIC and the circle of inhibition experiments mentioned above.

### 3.7. Effect of Composite Coating on the Leakage of Serratia marcescens and Pseudomonas syringae

**Figure 7:**
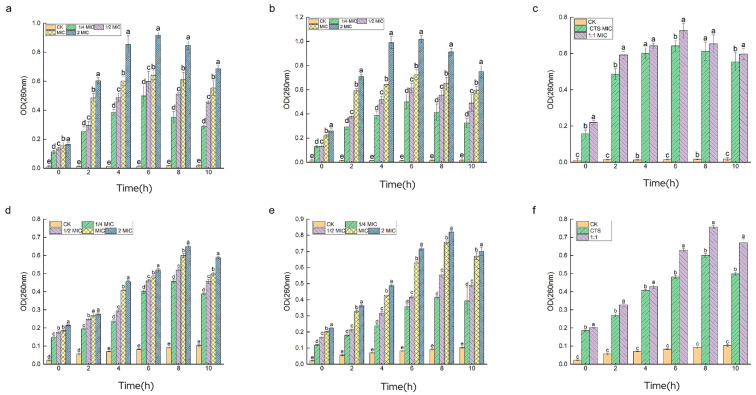
Plot of changes in nucleic acid leakage from *Serratia marcescens* and *Pseudomonas syringae*. Among them, (**a**–**c**) is *Pseudomonas syringae*, (**d**–**f**) is *Serratia marcescens*, and from left to right are CTS, composite coating solution (1:1), and MIC, respectively. Different lowercase letters indicate remarkable differences between groups at *p* ≤ 0.05.

**Figure 8:**
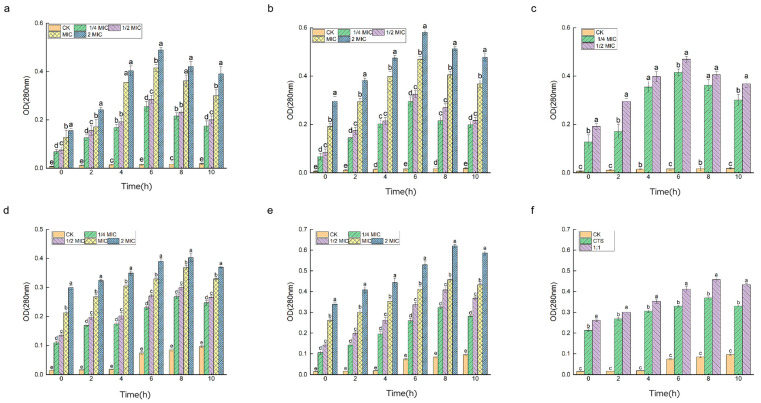
Plot of protein leakage changes in *Serratia marcescens* and *Pseudomonas syringae*. Among them, (**a**–**c**) is *Pseudomonas syringae*, (**d**–**f**) is *Serratia marcescens*, and from left to right are CTS, composite coating solution (1:1), and MIC, respectively. Different lowercase letters indicate remarkable differences between groups at *p* ≤ 0.05.

The cell membrane is a protective barrier for bacteria; if the cell membrane is disrupted, ions such as K^+^ will leak out first, followed by macromolecules such as DNA and RNA, etc. Cell contents with strong absorption values at 260 nm and 280 nm can be used to characterize the disruption of the cell membrane [21]. As shown in Figure 7, different concentrations of both drugs showed significant bacteriostatic activity against the two test bacteria, and their absorbance values at 260 nm wavelength were significantly greater than those of the control group, which increased significantly with the prolongation of the action time of the drug treatments. This indicates that CTS and composite coating solution can cause changes in the permeability of the cell membrane of *Serratia marcescens* and *Pseudomonas syringae*, which can cause the leakage of large molecules, such as DNA and RNA, and make the cell membrane incomplete, so the target of its inhibitory effect is on the cell membrane. A comparison of Figure 8a–c and Figure 8d–f shows that the drug with a concentration of 2 × MIC had the most significant DNA coloration effect in the two experimental bacteria. The control group of *Pseudomonas syringae* was close to 0, while the control group of *Serratia marcescens* had a slight increase in OD_260_ values; the treatment group of *Pseudomonas syringae* had significantly higher OD values, this indicates that *Pseudomonas syringae* had more severe leakage of cell contents and was more sensitive to drug treatment, a result which is consistent with the above experimental results.

Figure 8 indicates that the changes in intracellular protein release are caused by cell membrane rupture. The OD_280_ values of the samples from all experimental groups increased with increasing exposure time, the overall trend was similar to that of Figure 7, and the rate of increase in optical density at 260/280 nm showed a good dose-dependence in all cases. This indicated that there was a loss of proteins and destruction of double stranded DNA. Moreover, there was a good linear correlation between the values at OD_280_ nm and the different concentrations of the two drugs.

### 3.8. Bacteriostasis Experiment on Cherry Tomatoes

**Figure 9:**
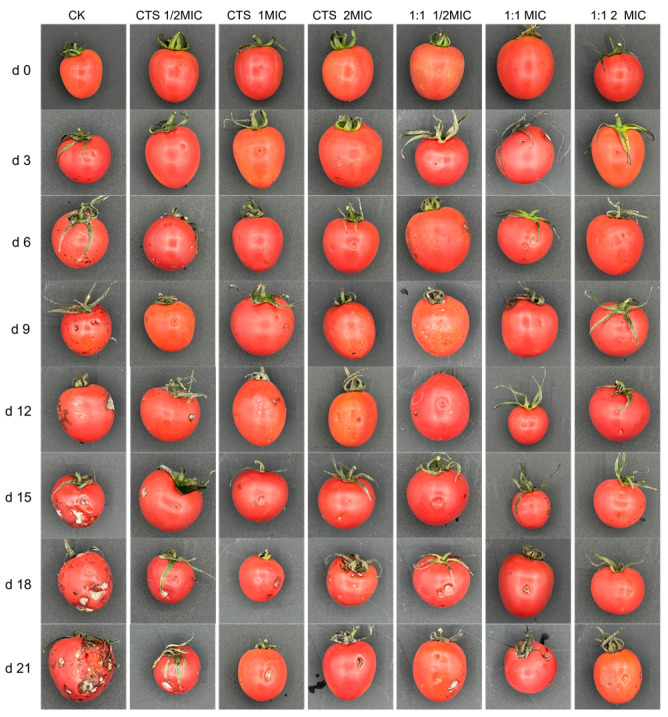
Effect of water-soluble CTS/CUR coatings on picked cherry tomato inoculated with *Pseudomonas syringae* was studied.

**Figure 10:**
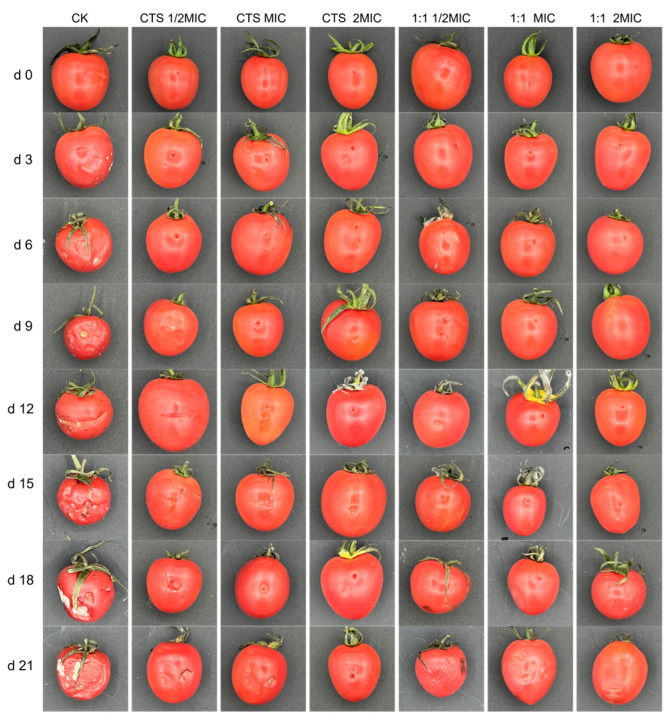
Effect of water-soluble CTS/CUR coatings on picked cherry tomato inoculated with *Serratia marcescens* was studied.

To confirm the bacteriostatic efficacy of the coating solution, the effect of CTS/CUR composite coatings on the resistance of post-harvest cherry tomatoes to *Serratia marcescens* and *Pseudomonas syringae* was further investigated. Figure 9 and Figure 10 demonstrate that the composite coating (1:1) had the most effective inhibitory effect on *Serratia marcescens* and *Pseudomonas syringae*. The appearance of cherry tomatoes inoculated with *Pseudomonas syringae* is depicted in Figure 9. The fruits of control group showed a similar appearance on the 3rd day of storage. On the 6th day, the 1/2 × MIC sample had mild symptoms. In addition, the incidence rate increased with the increase in storage time, and spots were clearly observed on the control and 1/2 × MIC samples. Figure 10 exhibits the appearance of cherry tomatoes inoculated with *Serratia marcescens*; the symptoms of tomato soft rot disease increased during storage. The fruits of the control group showed a similar appearance on the 3rd day of storage. On the 9th day, mild symptoms appeared on the 1/2 × MIC sample. In addition, the incidence rate increased with the increase in storage time, and obvious decay was clearly observed in the control and 1/2 × MIC samples.

## 4. Discussion

In our study, the results of the circle of inhibition experiments showed that both the CTS and the composite coating solution had significant circles of inhibition against the two test organisms, and the curdlan coating had a weak or no direct inhibitory effect, with the most pronounced effect on *Pseudomonas syringae*. The MIC and MBC determination tests determined the MIC and MBC of our material against two pathogenic bacteria, and combined with the results of the circle of inhibition experiments, it can be concluded that the antibacterial effect of the composite coating solution is stronger than that of the single CTS solution. The inhibitory effect of propyl gallate treatment of cherry tomatoes on *Serratia marcescens* was studied by Zhou J [28]. The results showed that the peel began to wrinkle on the 6th, and in contrast to their findings, the composite-coated-treated cherry tomatoes did not show peel wrinkling until the 15th day and were free of disease spots. In contrast to the study by Fan G [29], the composite coating treatment resulted in the leakage of large amounts of contents and more severe damage to the bacterial biofilm. Combined with our previous demonstration of the inhibitory effect of the composite coating on *Alternaria* and *Botrytis cinerea*, it shows that the composite coating is effective in preventing the invasion of microorganisms. The results of the determination of the effect on the bacterial growth curve after the action of the drugs showed that the CTS and the composite coating solution can change the normal growth cycle of bacteria, which is manifested in the change of the shape of the normal growth curve. When adding different concentrations of drug treatment, it delayed the arrival of the logarithmic period of the bacterial colony or hindered the growth of bacteria in varying degrees, which disrupted the general law of bacterial growth. When the concentration of the drug was 2 × MIC, the growth of the bacteria was completely inhibited or the bacteria were completely killed [30].

The cell membrane, also known as the cytoplasmic membrane, is a semi-permeable membrane that adheres closely to the inner side of the cell wall and is mainly composed of phospholipids and proteins. It is an important guarantee for maintaining the stability of the internal environment of cells and also an important structure for exchanging material and energy between cells and the outside world [31]. Nowadays, research has shown that many antibacterial and bactericidal agents achieve their antibacterial and bactericidal goals by destroying the cytoplasmic membrane of microorganisms [32]. Changes in conductivity can be used to illustrate the effect of drugs on the bacterial cell membrane. It was found that after treatment with drugs, the value of extracellular conductivity increased significantly within a short time, and as the concentration of drug action increased, the conductivity value also increased. This indicates that the cell membrane of bacteria is disrupted after treatment, which leads to bacterial inhibition. The destruction of the cytoplasmic membrane becomes more serious with the increase in the concentration of the drug, which leads to more leakage of ions, so that the cell membrane loses its important functions, which can cause the death of the bacterium [33]. To further illustrate the impact on the bacterial wall membrane system, normal bacterial cells and treated bacterial cells were observed under scanning electron microscopy. From the scanning electron microscopy images, we can clearly see that many cell surfaces appeared obvious folds, while the wall membrane was invaginated. On the basis of the previous experiments, PI fluorescence staining of bacterial cells was carried out, and it was found that the rate of breakage of the bacterium increased significantly with the increase in the concentration of the drug, which indicated the strong concentration dependence of the two drugs, and it also proved the destructive effect of the CTS and the composite coating solution on the cell membrane. The test results showed that no red fluorescence appeared in the blank group, indicating that the cell membrane of the normal state bacterium was intact and PI could not enter the cell for coloring, while red fluorescence could be observed after treatment, and the higher the concentration, the more and the stronger the red fluorescence was observed. This phenomenon indicates that the cell membrane was broken after treatment, and PI entered the cell to bind with DNA and emit red fluorescence, while the greater the concentration, the more the organisms whose cell membrane was broken.

The above results indicate that CTS and composite coating solutions first act on the cell wall and membrane during the antibacterial and bactericidal process, so that the morphology and integrity of cell wall and membrane was destroyed. Once the bacterial wall and membrane are destroyed, the bacterial environment will be imbalanced and the bacteria cannot carry out their normal material and energy metabolism, meaning they will gradually die [34]. Furthermore, some studies have shown that bacteriostatic fungicides can affect the expression of bacterial proteins and the synthesis of bacterial DNA, and some of them can act directly on DNA. The changes in OD_260_ and OD_280_ values can be used to illustrate the effect of pharmaceuticals on the leakage of bacterial DNA and proteins. Additionally, it was found that after the action of the pharmaceuticals, the value of OD_280_ increased significantly, which indicated that a large number of proteins leaked out of the pharmaceuticals after the action of the pharmaceuticals. The experiments on DNA also showed similar results, and the appearance of this result may be due to the fact that CTS and composite coating solution can cause changes in the permeability of the cell membrane of *Serratia marcescens* and *Pseudomonas syringae*,. This change can cause the leakage of large molecules, such as DNA and RNA, and the cell membrane becomes incomplete.

## 5. Conclusions

In this study, a novel edible composite coating that incorporates CUR into CTS coating solution was utilized to inhibit *Serratia marcescens* and *Pseudomonas syringae*, and the inhibition mechanism was analyzed. These results showed that this composite coating had a significant inhibitory effect on *Serratia marcescens* and *Pseudomonas syringae* and allowed the cell membranes of these two test bacteria to break down, causing the contents of the bacterial cells to leak out in large quantities and the bacterial morphology to alter. The CTS and composite coating solution effectively maintained the postharvest quality of cherry tomatoes and has the potential to significantly extend the shelf life of cherry tomatoes. Moreover, both CTS and CUR are inexpensive and can be used for large-scale production of composite coating. Therefore, they can be used widely of the preservation of fruits and vegetables.

## Figures and Tables

**Table 1:** MIC and MBC of CTS and CTS/CUR composite coating.

Strains	Water-Soluble Chitosan (μg/mL)		Composite Coating (1:1) (μg/mL)	
	MIC	MBC	MIC	MBC
*Serratia marcescens*	23.438	46.875	15.000	30.000
*Pseudomonas syringae*	18.750	37.500	11.719	23.438

## Data Availability

The original contributions presented in the study are included in the article, further inquiries can be directed to the corresponding author.
